# Microbiome Structure and Function in Woodchip Bioreactors for Nitrate Removal in Agricultural Drainage Water

**DOI:** 10.3389/fmicb.2021.678448

**Published:** 2021-08-06

**Authors:** Arnaud Jéglot, Joachim Audet, Sebastian Reinhold Sørensen, Kirk Schnorr, Finn Plauborg, Lars Elsgaard

**Affiliations:** ^1^Department of Agroecology, Aarhus University, Aarhus, Denmark; ^2^Centre for Water Technology (WATEC), Aarhus University, Aarhus, Denmark; ^3^Department of Bioscience, Aarhus University, Silkeborg, Denmark; ^4^Novozymes A/S, Kongens Lyngby, Denmark

**Keywords:** woodchip bioreactor, drainage water, environmental remediation, denitrification, nitrate, microbial diversity, metagenomics

## Abstract

Woodchip bioreactors are increasingly used to remove nitrate (NO_3_^–^) from agricultural drainage water in order to protect aquatic ecosystems from excess nitrogen. Nitrate removal in woodchip bioreactors is based on microbial processes, but the microbiomes and their role in bioreactor efficiency are generally poorly characterized. Using metagenomic analyses, we characterized the microbiomes from 3 full-scale bioreactors in Denmark, which had been operating for 4–7 years. The microbiomes were dominated by *Proteobacteria* and especially the genus *Pseudomonas*, which is consistent with heterotrophic denitrification as the main pathway of NO_3_^–^ reduction. This was supported by functional gene analyses, showing the presence of the full suite of denitrification genes from NO_3_^–^ reductases to nitrous oxide reductases. Genes encoding for dissimilatory NO_3_^–^ reduction to ammonium were found only in minor proportions. In addition to NO_3_^–^ reducers, the bioreactors harbored distinct functional groups, such as lignocellulose degrading fungi and bacteria, dissimilatory sulfate reducers and methanogens. Further, all bioreactors harbored genera of heterotrophic iron reducers and anaerobic iron oxidizers (*Acidovorax*) indicating a potential for iron-mediated denitrification. Ecological indices of species diversity showed high similarity between the bioreactors and between the different positions along the flow path, indicating that the woodchip resource niche was important in shaping the microbiome. This trait may be favorable for the development of common microbiological strategies to increase the NO_3_^–^ removal from agricultural drainage water.

## Introduction

Leaching of nitrate (NO_3_^–^) from agricultural soils to aquatic ecosystems is a growing environmental concern due to the globally increasing use of nitrogen (N) fertilizers in agriculture (Howarth et al., 2002). An effective way to mitigate NO_3_^–^ leaching is to treat agricultural drainage water before it reaches recipient waters, e.g., by use of woodchip bioreactors (Schipper et al., 2010b). In such facilities, woodchips provide the organic carbon (C) substrates and electron donors for anaerobic microorganisms that convert NO_3_^–^ to gaseous N species, thereby mitigating the N input to aqueous ecosystems. However, the NO_3_^–^ removal efficiency varies among bioreactors and may not always reach the environmental goals (Carstensen et al., 2020). Optimization of woodchip bioreactors has focused mainly on abiotic factors influencing NO_3_^–^ removal, such as dimensioning, hydraulic residence time (HRT), and physical matrix composition of the bioreactors (Cameron and Schipper, 2010; Chun et al., 2009). Yet, even under optimized conditions, NO_3_^–^ removal varies between woodchip bioreactors, indicating additional controls, most likely linked to the microbiology of these constructed ecosystems. Therefore, management of the bioreactor microbiomes to increase the NO_3_^–^ removal efficiency is an option that needs to be further explored and implemented (Grießmeier et al., 2017; Jang et al., 2019; Anderson et al., 2020). This necessitates a better understanding of the complex microbial populations and functional capacity of operating woodchip bioreactors that treat agricultural drainage water. So far, a number of studies have addressed the microbial composition of woodchip bioreactors under various conditions and at various scales using 16s rRNA and microbial cultivation methods (Grießmeier et al., 2017; Jang et al., 2019; Abdi et al., 2020; Hellman et al., 2021; Nordström et al., 2021). These studies allowed to link a number of factors, such as wood type, NO_3_^–^ concentration, and pesticide contamination with the presence and activity of specific microbes. The previous studies generally highlighted the importance of the phylum *Proteobacteria* in the bioreactor ecosystems, but provided limited insight on the entire woodchip microbiome.

Here we used metagenomics to obtain a more complete overview of the taxonomic composition and functional potential of woodchip bioreactor microbiomes. We report the microbiome composition at a given time point in three stable operating full-scale woodchip bioreactors (BR1-BR3), where NO_3_^–^ removal efficiencies were previously monitored (Bruun et al., 2016; Carstensen et al., 2019; Audet et al., 2021) and where concurrent metadata on water chemistry were available. The aim was to determine if the microbiomes differed among the bioreactors due to different design parameters and location in different agricultural settings. Because of a close physical location and similarity in design, the microbiomes of BR1 and BR2 were hypothesized to be more similar to each other than to BR3, which had a smaller ratio of bioreactor area to drained area and achieved greater NO_3_^–^ removal efficiency (Audet et al., 2021). Our second aim was to address how the microbiome and functional potential differed along the flow path within the bioreactors due to gradual NO_3_^–^ depletion, which may regulate the potential of anaerobic respiration based on other terminal electron acceptors, such as sulfate (SO_4_^2–^) and carbon dioxide (CO_2_) with resulting environmental losses of hydrogen sulfide (H_2_S) and methane (CH_4_), respectively.

## Materials and Methods

### Bioreactors and Woodchip Sampling

**FIGURE 1:**
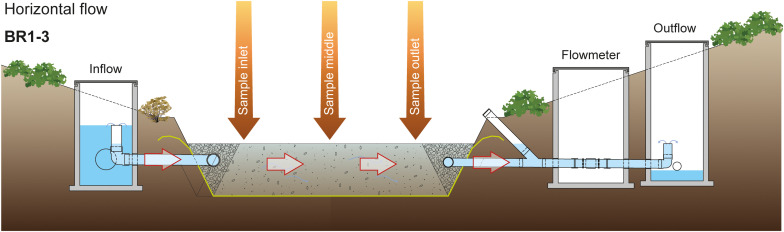
Sketch of the woodchip bioreactors (BR). From the inflow, where sedimentation tanks are located, the water goes through a matrix composed of woodchips (BR3) or a mix of woodchips and seashells (BR1 and BR2). The water goes out through a flow meter and an oxygenation device.

Woodchips were sampled from three woodchip bioreactors in Denmark (2019-11-20), designed conceptually as shown in Figure 1. The annual mean NO_3_^–^ concentrations at the inlet of BR1, BR2 and BR3 in 2019 were 49, 52 and 61 mg NO_3_^–^ L^–1^, respectively, and annual NO_3_^–^ removal efficiencies were typically 26–56% at BR1 and BR2 and 40–70% at BR3 (Carstensen et al., 2019; Audet et al., 2021). The bioreactors had horizontal flow design (Figure 1) and received subsurface drainage water from agricultural fields mainly with annual cropping systems. BR1 and BR2 (56.214°N, 9.743°E) had dimensions of 10 × 10 × 1.2 m (W × L × D) and were filled with a matrix of willow woodchips (2–32 mm diameter; Ny Vraa Bioenergi I/S, Denmark) mixed with seashells (Danshells A/S, Denmark). The woodchip/seashell ratio (v/v) was 50:50 for BR1 and 75:25 for BR2. Seashells were used as a material to improve the physical matrix stability and enhance water conductivity (Bruun et al., 2016). Dimensions of BR3 (55.988°N, 10.081°E) were 8 × 31 × 1.2 m and it was filled with willow woodchips. At all sites, the woodchip matrix was water saturated, except for the top 0.2 m, which remained unsaturated and was exposed to free air (i.e., the bioreactors were not covered by a layer of soil). BR1 and BR2 had been operating since 2012 (Carstensen et al., 2019; Hoffmann et al., 2019) and BR3 had been operating since 2015. The ratio between the bioreactor area and the drained agricultural area was 1:1300 for BR1 and BR2 and 1:830 for BR3.

Woodchip materials for metagenomic analyses were collected at three positions along the flow path in each bioreactor, i.e., near the inlet, middle and outlet (Figure 1). The positions were about 2.5, 5, and 7.5 m from the water inlet at BR1 and BR2 and about 8.5, 14.5, and 22.5 m from the water inlet at BR3. At each position, a composite sample was obtained by pooling three subsamples (taken across the bioreactor) from the upper 0.3 m of the water-saturated woodchip layer (i.e., sampled at the depth interval from ca. 0.2–0.5 cm below the bioreactor surface). The samples were handled using sterile gloves and immediately transferred to zip-lock plastic bags, transported in a cooling box, and stored at −20°C (2 days) before analysis.

### Metagenomic Analyses and Microbial Diversity

Metagenomic analyses were performed after mixing the composite samples to obtain a representative woodchip subsample of 15–20 g. The woodchip material was placed in 50-mL test tubes with 35 mL of milliQ water and shaken overnight (150 rev min^–1^, 20°C). After settling, the supernatant was recovered and centrifuged to harvest the microorganisms (14,000 *g*, 10 min). The pellets were recovered and treated with lysozyme (Sigma Aldrich, United States) to facilitate cell lysis. DNA was extracted as previously described using QIAamp kit for QIACUBE (QIAGEN, Germany, cat no. 51126) and sequenced using an Illumina MiSeq system (Petersen et al., 2020). The sequencing depth was between 18 and 1611 mega base pairs (Mbp). The fastq sequences of the metagenomes (Supplementary Table 1) were uploaded on the metagenomics RAST (MG-RAST) server (Meyer et al., 2008) and analyzed using default parameters with a maximum E value of 10^–5^, minimum identity cut-off value of 60%, and minimum alignment length of 15 bp (Randle-Boggis et al., 2016). The sequences were compared with the Reference Sequence database (O’Leary et al., 2016) for taxonomical identification and with the Kyoto Encyclopedia of Genes and Genomes Orthology database (Kanehisa et al., 2004) for identification of functional genes associated with nitrate reduction (Zumft, 1997), including dissimilatory nitrate reduction to ammonium (DNRA) and denitrification (Supplementary Table 2). The genes chosen to assess denitrifying potential in the samples were markers of larger complexes as described in Kraft et al. (2011). More specifically, *narG* was chosen as marker of the abundance of nitrate reductase related to the membrane bound complex NarGHI; *napA* was marker for the periplasmic nitrate reductase encoded by the gene cluster *napABCDE*; *nir*, representing the sum of *nirS* and *nirK*, was marker for nitrite (NO_2_^–^) reductase; *norB* was marker for the membrane bound nitric oxide (NO) reductase gene; and *nosZ* was marker for the periplasmic nitrous oxide (N_2_O) reductase.

Unconstrained non-metric multidimensional scaling (NMDS) and constrained canonical analysis of principal coordinates (CAP), both using Bray-Curtis distance, were used to analyze the dissimilarity in taxonomic composition and abundance of the identified microbial genera between the bioreactors and between the different positions along the flow path of the bioreactors (Anderson and Willis, 2003; Oksanen et al., 2020). The data was rarefied using the function *vegdist* from the R package *vegan* (Oksanen et al., 2020) before conducting CAP (function *capscale*) using the same R package and NMDS with the function *nmds* from the R package *ecodist* (Goslee and Urban, 2007). These two analyses focused on variations along the flow path (from inlet to outlet) and between different woodchip bioreactors. Also, total species richness (*S*), Shannon diversity (*H*′) and Pielou evenness (*J*) were used as ecological indices of similarity (Pielou, 1975) and tested for differences between the bioreactors and between the different positions along the flow path of the bioreactors using the procedure for two-way ANOVA analysis without replication (Zar, 2010) and reporting of *p*-values (Hurlbert et al., 2019). All analyses were performed using R version 3.6.1 (R Development Core Team, 2020) including tests of normal distribution and homoscedasticity.

### Environmental Metadata

Sampling and analyses of environmental metadata and water chemistry at the bioreactor inlets and outlets were conducted as described in Audet et al. (2021). The bioreactors were continuously monitored and the environmental metadata retrieved for this study represented a period from within 10 days before to 1 day after the time of woodchip sampling for metagenomic analyses.

## Results

### Microbiome Composition and Functional Groups

The microbiomes of the woodchip bioreactors were dominated by the phylum *Proteobacteria* (70–91%) with *Pseudomonas* as the most abundant genus, ranging from a relative abundance of 26% at the middle of BR1 to 61% at the outlet of BR3 (Supplementary Figure 1). Two other core taxa in the bioreactors were *Actinobacteria* and *Firmicutes* (Supplementary Figure 1). The high abundance of *Actinobacteria* was driven by multiple genera, but mainly by the genus *Cellulomonas*. The phylum *Firmicutes* was mainly represented by the genera *Bacillus* and *Exiguobacterium*.

**FIGURE 2:**
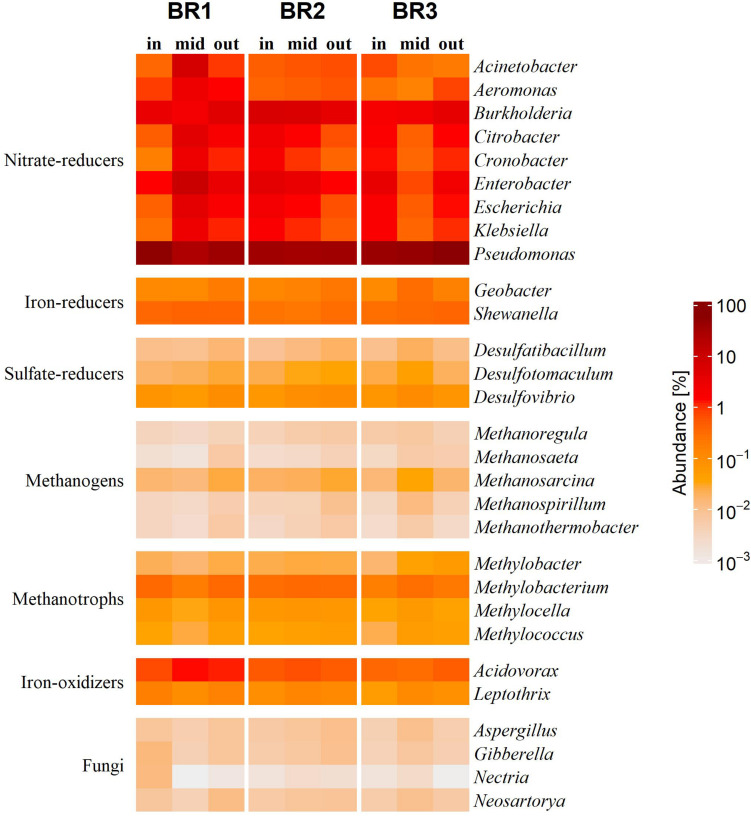
Heatmap of the relative abundance of microbial genera and functional metabolic groups in the woodchip bioreactors BR1, BR2, and BR3 close to the zones of inlet (in), middle (mid) and outlet (out) of the agricultural drainage water flow. The results are presented as the relative abundance of the hits attributed to each genus in percent of the total amount of hits obtained from the sample.

The functional metabolic groups in the bioreactors were dominated by heterotrophic NO_3_^–^ reducers, which in addition to *Pseudomonas* were represented by, e.g., *Burkholderia* and *Enterobacter* (Figure 2). The second most abundant metabolic groups were heterotrophic ferric iron (Fe^3+^) reducers (*Geobacter* and *Shewanella*) and ferrous iron (Fe^2+^) oxidizers with *Acidovorax* as the dominating genus. Dissimilatory sulfate-reducers were represented mainly by *Desulfovibrio* and endospore-forming *Desulfotomaculum* (Figure 2). Archaea mediating both hydrogenotrophic and acetoclastic methanogenesis were found in all bioreactors, with *Methanosarcina* as the dominating genus. Aerobic methanotrophs and fungi were also found in all samples, with fungi dominated by the phylum *Ascomycota*, especially the genus *Aspergillus* in both asexual and sexual (*Neosartorya*)states.

### Microbiome Diversity

**FIGURE 3:**
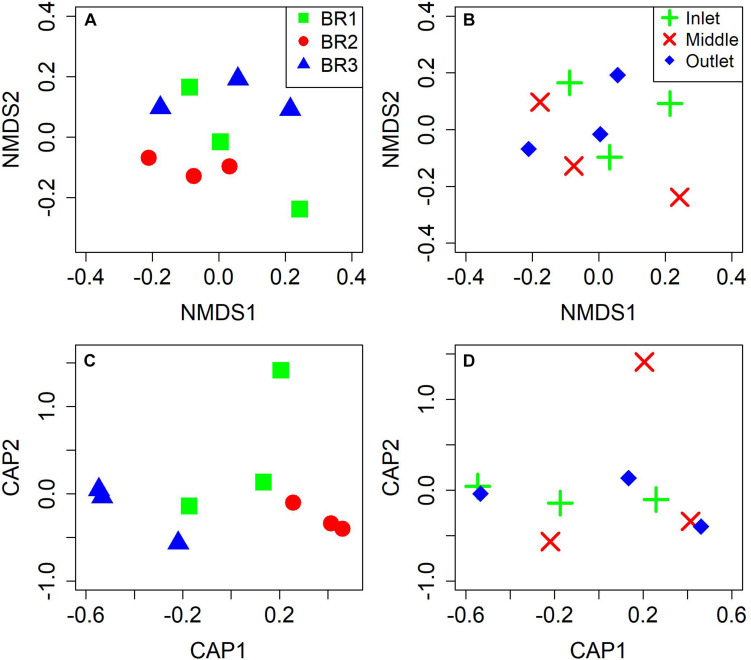
Plots of non-metric multidimensional scaling (NMDS) and canonical analysis of principal coordinates (CAP) using Bray-Curtis distance based on taxonomic diversity and abundance for the three bioreactors **(A,C)** and for the three zones of woodchip sampling **(B,D)**.

The microbiomes from the nine bioreactor samples were composed of 1,930 ± 180 identified species (*S*) with diversity indices (*H*′) of 4.28 ± 0.35 and evenness indices (*J*) of 0.57 ± 0.05 (mean ± 95% confidence interval) (Supplementary Table 3). Two-way ANOVA indicated high similarity of the ecological indices between bioreactors (*S*, *p* = 0.43; *H*′, *p* = 0.06; *J*, *p* = 0.11) and notably along the flow path of the bioreactors (*S*, *p* = 0.56; *H*′, *p* = 0.27; *J*, *p* = 0.40). NMDS and CAP analyses likewise indicated similar microbial compositions (Figure 3) with ANOVA test for CAP showing *p* = 0.25 for differences between bioreactors and *p* = 0.96 for differences between the positions in the bioreactors.

### Functional Genes

**FIGURE 4:**
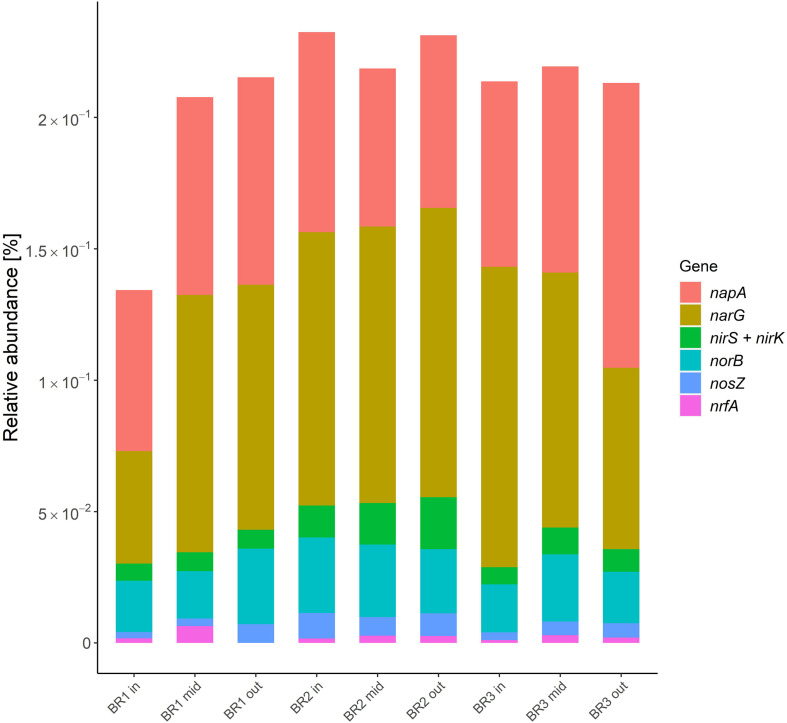
Functional genes of denitrification and dissimilatory nitrate reduction to ammonium (*nrfA*) in samples from bioreactors BR1, BR2, and BR3 at the zones close to the inlet (in), middle (mid) and outlet (out) of the agricultural drainage water flow. The results are presented as the relative abundance of the hits attributed to each gene in percent of the total amount of hits obtained from the samples with the KO database. Encoding genes: *Nar* and *Nap*, nitrate reductase; *nir*, nitrite reductase; *nor*, nitric oxide reductase; *nosZ*, nitrous oxide reductase; *nrfA*, nitrite reductase by formate (see Supplementary Table 2).

The relative abundance of functional nitrate reduction and denitrification genes was rather similar across the woodchip samples (Figure 4), and always with negligible contribution from *nrfA*, which is involved in DNRA (10 times lower relative abundance than *nir*). Nitrate reductase genes (*narG* and *napA*) associated with reduction of NO_3_^–^ to NO_2_^–^ were found in highest relative abundance (Figure 4), and exceeded the abundance of NO_2_^–^ reductases (*nir*) by an order of magnitude. The *nosZ* genes for N_2_O reductase were generally found in lowest relative abundance, except when compared to *nrfA*. Ratios of *nir* to *nosZ* (*nir/nosZ*) ranged from 1.0 to 2.7 with a mean of 2.0 ± 0.6 (mean ± standard deviation, *n* = 9), indicating comparable numbers of genes coding for enzymes related to production and consumption of N_2_O.

### Environmental Metadata

Based on the environmental metadata (Supplementary Table 4) there were clear differences between the bioreactors. First, BR3 had a much higher HRT than BR1 and BR2, which was influenced by the hydrological settings and the bioreactor volumes. Linked to the higher HRT for BR3, this bioreactor showed lower total N and NO_3_^–^ concentrations at the outlet, as well as higher CH_4_ production as compared with BR1 and BR2. In addition, BR3 reduced the concentrations of N_2_O in the inlet water from 17.8 to 2.3 μg N_2_O-N L^–1^, whereas N_2_O concentrations were lower at the inlet of BR1 and BR2 (4.0 μg N_2_O-N L^–1^), but increased toward the outlet (5.3–12.2 μg N_2_O-N L^–1^).

## Discussion

### Functional Groups

The prevalence of the genus *Pseudomonas*, combined with low abundance of DNRA genes, supported previous studies showing heterotrophic denitrification as the main NO_3_^–^ converting process in woodchip bioreactors (Schipper et al., 2010a). Thus, pseudomonads are known to thrive in soils and freshwater environments and contribute to denitrification as facultative anaerobes, since many species possess all the genes associated with complete denitrification (Lalucat et al., 2006). The relative abundance of functional denitrification genes showed a similar pattern across and within the bioreactors (Figure 4), and indicated relatively low *nir*/*nosZ* ratios, consistent with a low risk of N_2_O emissions (Conthe et al., 2019). This was in agreement with field measurements of dissolved and gaseous N_2_O fluxes from the three mature woodchip bioreactors, which showed a mean N_2_O-N emission factor of 0.6% of NO_3_-N removal (Audet et al., 2021). In studies of lab-scale woodchip bioreactors, ratios of *nir*/*nosZ* were initially high (> 10), but decreased after 6 months (Hellman et al., 2021), which supported that the genetic potential for N_2_O reduction develops toward generally low emissions from denitrifying bioreactors (Aalto et al., 2020).

The genera *Geobacter* and *Shewanella*, found in all bioreactors, are versatile heterotrophs that may oxidize a range of organic substrates under anoxic conditions by dissimilatory reduction of Fe^3+^ to Fe^2+^ (Bird et al., 2011). In the bioreactors, the iron reducers co-existed with *Acidovorax*, known to comprise Fe^2+^ oxidizing anaerobes that may utilize NO_3_^–^ or NO_2_^–^ as electron acceptor (Liu et al., 2019), thereby potentially contributing to iron-mediated denitrification (Bryce et al., 2018). Thus, microbial groups were identified, which could drive a dynamic cycling between Fe^3+^ and Fe^2+^ in the bioreactors, fueled by electrons from woodchip C and leading to denitrification. Such denitrification mediated by iron cycling has been reported for nitrate–rich groundwater settings (Hill, 2019) and wetland soil where the process was suggested to be of ecological relevance (Petersen et al., 2020). However, it is uncertain to what extent the entire process would be microbially mediated, since abiotic denitrification may occur by chemical Fe^2+^ reactions with N intermediates, such as NO_2_^–^ (Klueglein and Kappler, 2013; Jones et al., 2015). Such chemodenitrification has been suggested to increase the risk of N_2_O emissions from other C-rich ecosystems (Zhu-Barker et al., 2015), but so far, both the pathways, Fe availability, and ecological importance of iron-mediated denitrification in woodchip bioreactors remain to be documented.

The presence of dissimilatory sulfate-reducers in the woodchip bioreactors substantiated the risk of H_2_S formation, which has previously been indicated at high HRT when NO_3_^–^ is fully depleted (Carstensen et al., 2019). Likewise, the presence of methanogens supported previous reports of CH_4_ emissions from all three bioreactors (Bruun et al., 2017; Carstensen et al., 2019), with a higher production from BR3. Hence, the metabolic diversity of the bioreactors included anaerobes using SO_4_^2–^ or CO_2_ as electron acceptors, which thermodynamically would be favorable only after depletion of NO_3_^–^ (Thauer et al., 1977). Yet, there was no clear stratification of these metabolic types along the flow path of the bioreactors, indicating a latent potential of H_2_S and CH_4_ formation at times of complete NO_3_^–^ removal. Moreover, it was surprising to find similar abundances of methanogenic populations between the bioreactors since a higher abundance could have been expected in BR3, because of the higher HRT and CH_4_ production.

The omnipresence of fungal genera suggested a potential catabolic role related to degradation of wood-derived lignocellulose to simple C substrates for denitrification. The activity of fungi in denitrifying bioreactors may be restricted by low oxygen (O_2_) availability, but due to the inflow of oxygenated drain water, oxic conditions may prevail at least temporarily in the woodchip matrix at concentration gradients, which decrease along the flow path and depth in the bioreactors (Jéglot, unpublished results). So far, however, little is known about the life and ecological role of fungi in denitrifying woodchip bioreactors. Remarkably, and in addition a role in lignocellulose degradation, Aldossari and Ishii (2020) newly reported the occurrence of denitrifying fungi from a woodchip bioreactor in Minnesota (United States), where these organisms seemingly contributed directly to anaerobic N transformations, thus indicating a novel physiological and ecological role to be further examined.

Although fungi have been commonly associated with degradation of lignocellulose, an increasing number of bacterial enzymes have been identified, which are also able to degrade these compounds. *Proteobacteria*, *Firmicutes*, *Actinobacteria*, *Verrucomicrobia*, and *Bacteroidetes* have all been found to contain genera that may attack lignin structures (López-Mondéjar et al., 2019). Therefore, the relative importance of fungi and bacteria for lignocellulose degradation in woodchip bioreactors may depend on competition and environmental constraints that regulates the activity of the different groups. In a metagenomic and metatranscriptomic study of a denitrifying beech woodchip bioreactor in Germany, Grießmeier et al. (2021) found that bacteria contributed to the wood decomposition process to similar extent as fungi. Nevertheless, a general scheme was suggested where fungi in cellulose hydrolyzing biofilms on woodchip surfaces provided soluble labile C compounds as electron donors for denitrifying bacteria predominantly occurring in the planktonic phase of the bioreactor (Grießmeier et al., 2021). In the present study, the genus *Cellulomomas* was prevalent, which may produce starch-, xylan-, and cellulose-degrading enzymes under microaerobic or even anaerobic conditions (Stackebrandt and Schumann, 2014) and thereby contribute to initial woodchip degradation. Based on genome analyses of *Cellulomonas* sp. strain WB94, isolated from a woodchip bioreactor in Minnesota, Jang et al. (2019) recently reported the occurrence of denitrification genes in *Cellulomonas*, which indicated that this genus might contribute to both initial degradation of complex C compounds and complete C mineralization by denitrification in woodchip bioreactors.

### Microbiome Diversity

The bioreactors BR1 and BR2 deviated from BR3 in design, environmental location and characteristics of NO_3_^–^ removal efficiency (Carstensen et al., 2019; Audet et al., 2021). Yet, a high similarity of the microbial communities between the bioreactors and between the different positions within the bioreactors was found. This indicated (i) that difference in design parameters, and especially HRT, did not significantly influence the microbiome of the woodchip bioreactors, and (ii) that microorganisms transported in the drain water from the surrounding agricultural fields were either rather similar or did not have a strong impact on the microbiomes established in the bioreactors. A metagenomic study with more than 700 semi-aquatic bacterial communities, sampled over five orders of spatial distance, emphasized the general impact of resource niches over more stochastic effects in shaping the community-level signatures of bacterial communities (Pascual-García and Bell, 2020). Also, a strong role of substrate type in shaping the microbial community composition was indicated in studies of lab-scale denitrifying bioreactors based on sedge, barley straw and pine woodchips (Hellman et al., 2021). Therefore, full-scale willow woodchip bioreactors receiving nitrate-rich drainage water from agricultural fields could provide consistent resource niches for congruent microbiomes that develop across different bioreactors. If so, one could also expect that common strategies of microbial management (e.g., bioaugmentation or biostimulation) might be exploited across such woodchip bioreactors to increase the efficiency of NO_3_^–^ removal from agricultural drainage water.

Only few studies have tried to reveal how microbial communities fluctuate seasonally within individual field-scale denitrifying bioreactors. Grießmeier et al. (2021) indicated a stable microbial community composition (dominated by phylum Proteobacteria) over different seasons in a beech woodchip bioreactor, i.e., with minor influence from HRT and temperature changes. Yet, in another study, Jéglot et al. (2021) indicated divergent temperature responses of denitrifiers from a willow woodchip bioreactor, thus suggesting a potential adaptation of the woodchip microbiome to seasonal temperature changes. Thus, further studies with sampling at multiple time points for metagenomic and metatranscriptomic analyses are required to better characterize the microbial functioning of woodchip bioreactors in response to environmental temperature fluctuations.

## Conclusion

The present study showed high similarity in the microbiomes between bioreactors and between different positions within three operating full-scale woodchip bioreactors at a given time point, indicating minor importance of design parameters and location in different agricultural settings. Major microbial groups and genes in NO_3_^–^ reduction pathways were associated with denitrification with only minor contribution from DNRA. Functional diversity related to denitrification further included microbial lignocellulose degradation and bacterial Fe cycling, which are processes that should be further examined for their ecological role in efficient NO_3_^–^ removal from agricultural drainage water.

## Data Availability Statement

The datasets presented in this study can be found in online repositories. The names of the repository/repositories and accession number(s) can be found in the article/Supplementary Material.

## Author Contributions

AJ, LE, and JA designed the research. AJ and JA conducted the experiments and analyses. AJ, JA, SS, KS, FP, and LE wrote the manuscript. All authors contributed to the article and approved the submitted version.

## Conflict of Interest

The authors declare that the research was conducted in the absence of any commercial or financial relationships that could be construed as a potential conflict of interest.

## Publisher’s Note

All claims expressed in this article are solely those of the authors and do not necessarily represent those of their affiliated organizations, or those of the publisher, the editors and the reviewers. Any product that may be evaluated in this article, or claim that may be made by its manufacturer, is not guaranteed or endorsed by the publisher.

## Abbreviations

BP: base pair
BR: bioreactor
CAP: constrained canonical analysis of principal coordinates
DNRA: dissimilatory nitrate reduction to ammonium
NMDS: non-metric multidimensional scaling.

## References

[B1] Aalto S. L. Suurnakki S. von Ahnen M. Siljanen H. M. P. Pedersen P. B. Tiirola M. (2020). Nitrate removal microbiology in woodchip bioreactors: a case-study with full-scale bioreactors treating aquaculture effluents. *Sci. Total Environ.* 723:138093. 10.1016/j.scitotenv.2020.138093 32222508

[B2] Abdi D. E. Owen J. S. Jr. Brindley J. C. Birnbaum A. C. Wilson P. C. Hinz F. O. (2020). Nutrient and pesticide remediation using a two-stage bioreactor-adsorptive system under two hydraulic retention times. *Water Res.* 170:115311. 10.1016/j.watres.2019.115311 31783190

[B3] Aldossari N. Ishii S. (2020). Isolation of cold-adapted nitrate-reducing fungi that have potential to increase nitrate removal in woodchip bioreactors. *J. Appl. Microbiol.* 131 197–207. 10.1111/jam.14939 33222401

[B4] Anderson E. Jang J. Venterea R. Feyereisen G. Ishii S. (2020). Isolation and characterization of denitrifiers from woodchip bioreactors for bioaugmentation application. *J. Appl. Microbiol.* 129 590–600. 10.1111/jam.14655 32259336

[B5] Anderson M. J. Willis T. J. (2003). Canonical analysis of principal coordinates: a useful method of constrained ordination for ecology. *Ecology* 84 511–525. 10.1890/0012-9658(2003)084[0511:caopca]2.0.co;2

[B6] Audet J. Jéglot A. Elsgaard L. Maagard A. L. Sørensen S. R. Zak D. (2021). Nitrogen removal and nitrous oxide emissions from woodchip bioreactors treating agricultural drainage waters. *Ecol. Eng.* 169:106328. 10.1016/j.ecoleng.2021.106328

[B7] Bird L. J. Bonnefoy V. Newman D. K. (2011). Bioenergetic challenges of microbial iron metabolisms. *Trends Microbiol.* 19 330–340. 10.1016/j.tim.2011.05.001 21664821

[B8] Bruun J. Hoffmann C. C. Kjaergaard C. (2016). Nitrogen removal in permeable woodchip filters affected by hydraulic loading rate and woodchip ratio. *J. Environ. Qual.* 45 1688–1695. 10.2134/jeq2015.11.0583 27695766

[B9] Bruun J. Hoffmann C. C. Kjaergaard C. (2017). Convective transport of dissolved gases determines the fate of the greenhouse gases produced in reactive drainage filters. *Ecol. Eng.* 98 1–10. 10.1016/j.ecoleng.2016.10.027

[B10] Bryce C. Blackwell N. Schmidt C. Otte J. Huang Y.-M. Kleindienst S. (2018). Microbial anaerobic Fe(II) oxidation - ecology, mechanisms and environmental implications. *Environ. Microbiol.* 20 3462–3483. 10.1111/1462-2920.14328 30058270

[B11] Cameron S. G. Schipper L. A. (2010). Nitrate removal and hydraulic performance of organic carbon for use in denitrification beds. *Ecol. Eng.* 36 1588–1595. 10.1016/j.ecoleng.2010.03.010

[B12] Carstensen M. V. Hashemi F. Hoffmann C. C. Zak D. Audet J. Kronvang B. (2020). Efficiency of mitigation measures targeting nutrient losses from agricultural drainage systems: a review. *Ambio* 49 1820–1837. 10.1007/s13280-020-01345-5 32494964 PMC7502647

[B13] Carstensen M. V. Larsen S. E. Kjærgaard C. Hoffmann C. C. (2019). Reducing adverse side effects by seasonally lowering nitrate removal in subsurface flow constructed wetlands. *J. Environ. Manag.* 240 190–197. 10.1016/j.jenvman.2019.03.081 30933823

[B14] Chun J. A. Cooke R. A. Eheart J. W. Kang M. S. (2009). Estimation of flow and transport parameters for woodchip-based bioreactors: I. Laboratory-scale bioreactor. *Biosyst. Eng.* 104 384–395. 10.1016/j.biosystemseng.2009.06.021

[B15] Conthe M. Lycus P. Arntzen M. O. Ramos da Silva A. Frostegard A. Bakken L. R. (2019). Denitrification as an N_2_O sink. *Water Res.* 151 381–387. 10.1016/j.watres.2018.11.087 30616050

[B16] Goslee S. C. Urban D. L. (2007). The ecodist package for dissimilarity-based analysis of ecological data. *J. Stat. Softw.* 22:20893. 10.18637/jss.v022.i07

[B17] Grießmeier V. Bremges A. McHardy A. C. Gescher J. (2017). Investigation of different nitrogen reduction routes and their key microbial players in wood chip-driven denitrification beds. *Sci. Rep.* 7:17028. 10.1038/s41598-017-17312-2 29208961 PMC5716999

[B18] Grießmeier V. Wienhöfer J. Horn H. Gescher J. (2021). Assessing and modeling biocatalysis in field denitrification beds reveals key influencing factors for future constructions. *Water Res.* 188:116467. 10.1016/j.watres.2020.116467 33068909

[B19] Hellman M. Hubalek V. Juhanson J. Almstrand R. Peura S. Hallin S. (2021). Substrate type determines microbial activity and community composition in bioreactors for nitrate removal by denitrification at low temperature. *Sci. Total Environ.* 755:143023. 10.1016/j.scitotenv.2020.143023 33158531

[B20] Hill A. R. (2019). Groundwater nitrate removal in riparian buffer zones: a review of research progress in the past 20 years. *Biogeochemistry* 143 347–369. 10.1007/s10533-019-00566-5

[B21] Hoffmann C. C. Larsen S. E. Kjaergaard C. (2019). Nitrogen removal in woodchip-based biofilters of variable designs treating agricultural drainage discharges. *J. Environ. Qual.* 48 1881–1889. 10.2134/jeq2018.12.0442

[B22] Howarth R. Sharpley A. Walker D. (2002). Sources of nutrient pollution to coastal waters in the united states: implications for achieving coastal water quality goals. *Estuaries* 25 656–676. 10.1007/BF02804898

[B23] Hurlbert S. H. Levine R. A. Utts J. (2019). Coup de grâce for a tough old bull: “statistically significant” expires. *Am. Stat.* 73 352–357. 10.1080/00031305.2018.1543616

[B24] Jang J. Anderson E. L. Venterea R. T. Sadowsky M. J. Rosen C. J. Feyereisen G. W. (2019). Denitrifying bacteria active in woodchip bioreactors at low-temperature conditions. *Front. Microbiol.* 10:635. 10.3389/fmicb.2019.00635 31001220 PMC6454037

[B25] Jéglot A. Sørensen S. R. Schnorr K. M. Plauborg F. Elsgaard L. (2021). Temperature sensitivity and composition of nitrate-reducing microbiomes from a full-scale woodchip bioreactor treating agricultural drainage water. *Microorganisms* 9:1331. 10.3390/microorganisms9061331 34207422 PMC8235139

[B26] Jones L. C. Peters B. Lezama Pacheco J. S. Casciotti K. L. Fendorf S. (2015). Stable isotopes and iron oxide mineral products as markers of chemodenitrification. *Environ. Sci. Technol.* 49 3444–3452. 10.1021/es504862x 25683572

[B27] Kanehisa M. Goto S. Kawashima S. Okuno Y. Hattori M. (2004). The KEGG resource for deciphering the genome. *Nucleic Acids Res.* 32 D277–D280. 10.1093/nar/gkh063 14681412 PMC308797

[B28] Klueglein N. Kappler A. (2013). Abiotic oxidation of Fe(II) by reactive nitrogen species in cultures of the nitrate-reducing Fe(II) oxidizer *Acidovorax* sp. BoFeN1 - questioning the existence of enzymatic Fe(II) oxidation. *Geobiology* 11 180–190. 10.1111/gbi.12019 23205609

[B29] Kraft B. Strous M. Tegetmeyer H. E. (2011). Microbial nitrate respiration - genes, enzymes and environmental distribution. *J. Biotechnol.* 155 104–117. 10.1016/j.jbiotec.2010.12.025 21219945

[B30] Lalucat J. Bennasar A. Bosch R. García-Valdés E. Palleroni N. J. (2006). Biology of *Pseudomonas stutzeri*. *Microbiol. Mol. Biol. Rev.* 70 510–547. 10.1128/MMBR.00047-05 16760312 PMC1489536

[B31] Liu T. Chen D. Li X. Li F. (2019). Microbially mediated coupling of nitrate reduction and Fe(II) oxidation under anoxic conditions. *FEMS Microbiol. Ecol.* 95:fiz030. 10.1093/femsec/fiz030 30844067

[B32] López-Mondéjar R. Algora C. Baldrian P. (2019). Lignocellulolytic systems of soil bacteria: a vast and diverse toolbox for biotechnological conversion processes. *Biotechnol. Adv.* 37:107374. 10.1016/j.biotechadv.2019.03.013 30910513

[B33] Meyer F. Paarmann D. D’Souza M. Olson R. Glass E. M. Kubal M. (2008). The metagenomics RAST server - a public resource for the automatic phylogenetic and functional analysis of metagenomes. *BMC Bioinform.* 9:386. 10.1186/1471-2105-9-386 18803844 PMC2563014

[B34] Nordström A. Hellman M. Hallin S. Herbert R. B. (2021). Microbial controls on net production of nitrous oxide in a denitrifying woodchip bioreactor. *J. Environ. Qual.* 50 228–240. 10.1002/jeq2.20181 33270921

[B35] Oksanen J. Blanchet F. G. Friendly M. Kindt R. Legendre P. McGlinn D. (2020). *vegan: Community Ecology Package. R Package Version 2.5-7.* Available online at: https://CRAN.R-project.org/package=vegan (accessed October 12, 2020).

[B36] O’Leary N. A. Wright M. W. Brister J. R. Ciufo S. Haddad D. McVeigh R. (2016). Reference sequence (RefSeq) database at NCBI: current status, taxonomic expansion, and functional annotation. *Nucleic Acids Res.* 44 D733–D745. 10.1093/nar/gkv1189 26553804 PMC4702849

[B37] Pascual-García A. Bell T. (2020). Community-level signatures of ecological succession in natural bacterial communities. *Nat. Commun.* 11:2386. 10.1038/s41467-020-16011-3 32404904 PMC7220908

[B38] Petersen R. J. Liang Z. Prinds C. Jéglot A. Thamdrup B. Kjærgaard C. (2020). Nitrate reduction pathways and interactions with iron in the drainage water infiltration zone of a riparian wetland soil. *Biogeochemistry* 150 235–255. 10.1007/s10533-020-00695-2

[B39] Pielou E. C. (1975). *Ecological Diversity.* New York, NY: Wiley.

[B40] R Development Core Team (2020). *R: A Language and Environment for Statistical Computing.* Vienna: R Foundation for Statistical Computing.

[B41] Randle-Boggis R. J. Helgason T. Sapp M. Ashton P. D. (2016). Evaluating techniques for metagenome annotation using simulated sequence data. *FEMS Microbiol. Ecol.* 92:fiw095. 10.1093/femsec/fiw095 27162180 PMC4892715

[B42] Schipper L. A. Cameron S. C. Warneke S. (2010a). Nitrate removal from three different effluents using large-scale denitrification beds. *Ecol. Eng.* 36 1552–1557. 10.1016/j.ecoleng.2010.02.007

[B43] Schipper L. A. Robertson W. D. Gold A. J. Jaynes D. B. Cameron S. C. (2010b). Denitrifying bioreactors—An approach for reducing nitrate loads to receiving waters. *Ecol. Eng.* 36 1532–1543. 10.1016/j.ecoleng.2010.04.008

[B44] Stackebrandt E. Schumann P. (2014). “The family *Cellulomonadaceae*,” in *The Prokaryotes*, eds Rosenberg E. DeLong E. F. Lory S. Stackebrandt E. Thompson F. (Berlin: Springer), 163–184. 10.1007/978-3-642-30138-4_223

[B45] Thauer R. K. Jungermann K. Decker K. (1977). Energy conservation in chemotrophic anaerobic bacteria. *Bacteriol. Rev.* 41 100–180. 10.1128/mmbr.41.1.100-180.1977 860983 PMC413997

[B46] Zar J. H. (2010). *Biostatistical Analysis*, 5th Edn, Upper Sadle River, NJ: Prentice-Hall, Inc.

[B47] Zhu-Barker X. Cavazos A. R. Ostrom N. E. Horwath W. R. Glass J. (2015). The importance of abiotic reactions for nitrous oxide production. *Biogeochemistry* 126 251–267. 10.1007/s10533-015-0166-4

[B48] Zumft W. G. (1997). Cell biology and molecular basis of denitrification. *Microbiol. Mol. Biol. Rev.* 61 533–616. 10.1128/.61.4.533-616.1997 9409151 PMC232623

