# Supplementary material for: Microbiome Structure and Function in Woodchip Bioreactors for Nitrate Removal in Agricultural Drainage Water
Source: Front Microbiol. 2021 Aug 6;12:678448. doi: 10.3389/fmicb.2021.678448 (PMC8377596; doi:10.3389/fmicb.2021.678448)
Supplement: Supplementary file 1 [file Data_Sheet_1.docx]

Supplementary Material

**Supplementary Table 1.** DNA sample lengths and IDs on MG-RAST for the metagenomic samples sequenced from woodchips coming from bioreactors BR1, BR2 and BR3 at the inlet (in), at the middle (mid) and at the outlet (out) of the agricultural drainage water flow. To access the data, add the “MG-RAST ID” specified in the table to the following link: [http://mg-rast.org/linkin.cgi?metagenome=](http://mg-rast.org/linkin.cgi?metagenome=4440283.3)”MG-RAST ID”. The raw sequences have also been uploaded to ENA (European Nucleotide Archive), the accession numbers are provided in the table.

| Reactor | Zone | Length (base pairs) | MG-RAST ID | ENA Study | ENA Sample |
| --- | --- | --- | --- | --- | --- |
| BR1 | in | 1,029,235,182 | mgm4886144.3 | PRJEB41770 | ERS5924878 |
| BR1 | mid | 1,611,533,578 | mgm4886146.3 | PRJEB41770 | ERS5924879 |
| BR1 | out | 18,008,555 | mgm4886142.3 | PRJEB41770 | ERS5924880 |
| BR2 | in | 1,497,492,381 | mgm4886149.3 | PRJEB41770 | ERS5924881 |
| BR2 | mid | 1,372,085,113 | mgm4886148.3 | PRJEB41770 | ERS5924882 |
| BR2 | out | 1,249,780,127 | mgm4886143.3 | PRJEB41770 | ERS5924883 |
| BR3 | in | 1,083,912,863 | mgm4886147.3 | PRJEB41770 | ERS5924884 |
| BR3 | mid | 1,326,352,600 | mgm4886145.3 | PRJEB41770 | ERS5924885 |
| BR3 | out | 1,450,736,866 | mgm4886141.3 | PRJEB41770 | ERS5924886 |

**Supplementary Table 2.** Functional genes related to nitrate reduction and denitrification as retrieved from the Kyoto Encyclopedia of Genes and Genomes Orthology database (Kanehisa et al., 2004). *narG* was chosen as the marker for the enzymatic cluster NarGHI and *napA* as the marker for the gene cluster *napABCDE* .

| Enzyme name | Gene annotation |
| --- | --- |
| Nitrate reductase | *narG*: nitrate reductase 1, alpha subunit [EC:1.7.99.4]; |
|  | *napA:* periplasmic nitrate reductase [EC:1.7.99.4] |
| Nitrite reductase | *nir* (*nirS* and *nirK*): nitrite reductase (NO-forming) [EC:1.7.2.1] |
| Nitric oxide reductase | *norB*: nitric oxide reductase subunit B [EC:1.7.2.5] |
| Nitrous oxide reductase | *nosZ*: nitrous-oxide reductase [EC:1.7.2.4] |
| Nitrite reductase | *nrfA*: nitrite reductase by formate, cytochrome c-552 [EC:1.7.2.2] |

**Supplementary Table 3.** Total species richness (*S*), Shannon diversity index (*H'*), and Pielou evenness index (*J*) for the metagenomes sequenced from the bioreactors BR1, BR2, and BR3 at the zones of inlet, middle and outlet of the water flow. Means and 95% confidence intervals are shown across the nine samples.

| Bioreactor | Zone | Total species richness (*S*) |  | Shannon diversity index (*H'*) |  | Pielou evenness index (*J*) |
| --- | --- | --- | --- | --- | --- | --- |
| BR1 | Inlet | 1946 |  | 3.85 |  | 0.51 |
| BR1 | Middle | 2055 |  | 4.57 |  | 0.60 |
| BR1 | Outlet | 1322 |  | 4.57 |  | 0.64 |
| BR2 | Inlet | 2031 |  | 4.51 |  | 0.59 |
| BR2 | Middle | 2063 |  | 4.79 |  | 0.63 |
| BR2 | Outlet | 2015 |  | 4.66 |  | 0.61 |
| BR3 | Inlet | 1961 |  | 4.07 |  | 0.54 |
| BR3 | Middle | 2044 |  | 4.10 |  | 0.54 |
| BR3 | Outlet | 1970 |  | 3.41 |  | 0.45 |
| Mean  95% confidence interval | | 1930 |  | 4.28 |  | 0.57 |
|  |  | 180 |  | 0.35 |  | 0.05 |

**Supplementary Table 4.** Hydraulic retention time, temperature and water chemistry at the inlet and outlet of the woodchip bioreactors BR1, BR2, and BR3. Sampling for metadata analyses was performed within 10 days before to 1 day after woodchip sampling. Inlet and outlet refers to water samples collected before and after passage through the bioreactors, i.e., not the exact places of woodchip sampling designated as inlet and outlet. nd, not detectable. *High value on this day that does not reflect the generally low oxygen level at the outlet of this bioreactor.

| Property | Bioreactor | | | | |
| --- | --- | --- | --- | --- | --- |
|  | BR1 |  | BR2 |  | BR3 |
| Hydraulic retention time (h) | 12.9 |  | 13.4 |  | 450 |
| Temperature, inlet (°C) | 7.5 |  | 8.2 |  | 8.3 |
| pH, inlet | 7.4 |  | 7.4 |  | 7.2 |
| pH, outlet | 7.4 |  | 7.3 |  | 7.4 |
| Total nitrogen, inlet (mg N L^-1^) | 12.9 |  | 12.9 |  | 12.6 |
| Total nitrogen, outlet (mg N L^-1^) | 11.5 |  | 11.1 |  | 0.9 |
| Nitrate (NO_3_^-^), inlet (mg N L^-1^) | 11.4 |  | 11.4 |  | 11.7 |
| Nitrate (NO_3_^-^), outlet (mg N L^-1^) | 10.4 |  | 10.0 |  | 0.1 |
| Ammonium (NH_4_^+^), inlet (mg N L^-1^) | 0.01 |  | 0.01 |  | 0.02 |
| Ammonium (NH_4_^+^), outlet (mg N L^-1^) | nd |  | nd |  | 0.05 |
| Total organic carbon (TOC), inlet (mg C L^-1^) | 7.9 |  | 7.9 |  | 4.1 |
| Total organic carbon (TOC), outlet (mg C L^-1^) | 6.9 |  | 7.5 |  | 8.4 |
| Nitrous oxide (N_2_O), inlet (µg N L^-1^) | 4.0 |  | 4.0 |  | 17.8 |
| Nitrous oxide (N_2_O), outlet (µg N L^-1^) | 5.3 |  | 12.2 |  | 2.3 |
| Methane (CH_4_), inlet (µg C L^-1^) | 18 |  | 18 |  | 4 |
| Methane (CH_4_), outlet (µg C L^-1^) | 108 |  | 356 |  | 1440 |
| Oxygen (O_2_), inlet (mg O_2_ L^-1^) | 11.3 |  | 11.3 |  | 11.5 |
| Oxygen (O_2_), outlet (mg O_2_ L^-1^) | 0.5 |  | 0.1 |  | 8.2* |

**Supplementary Figure 1**


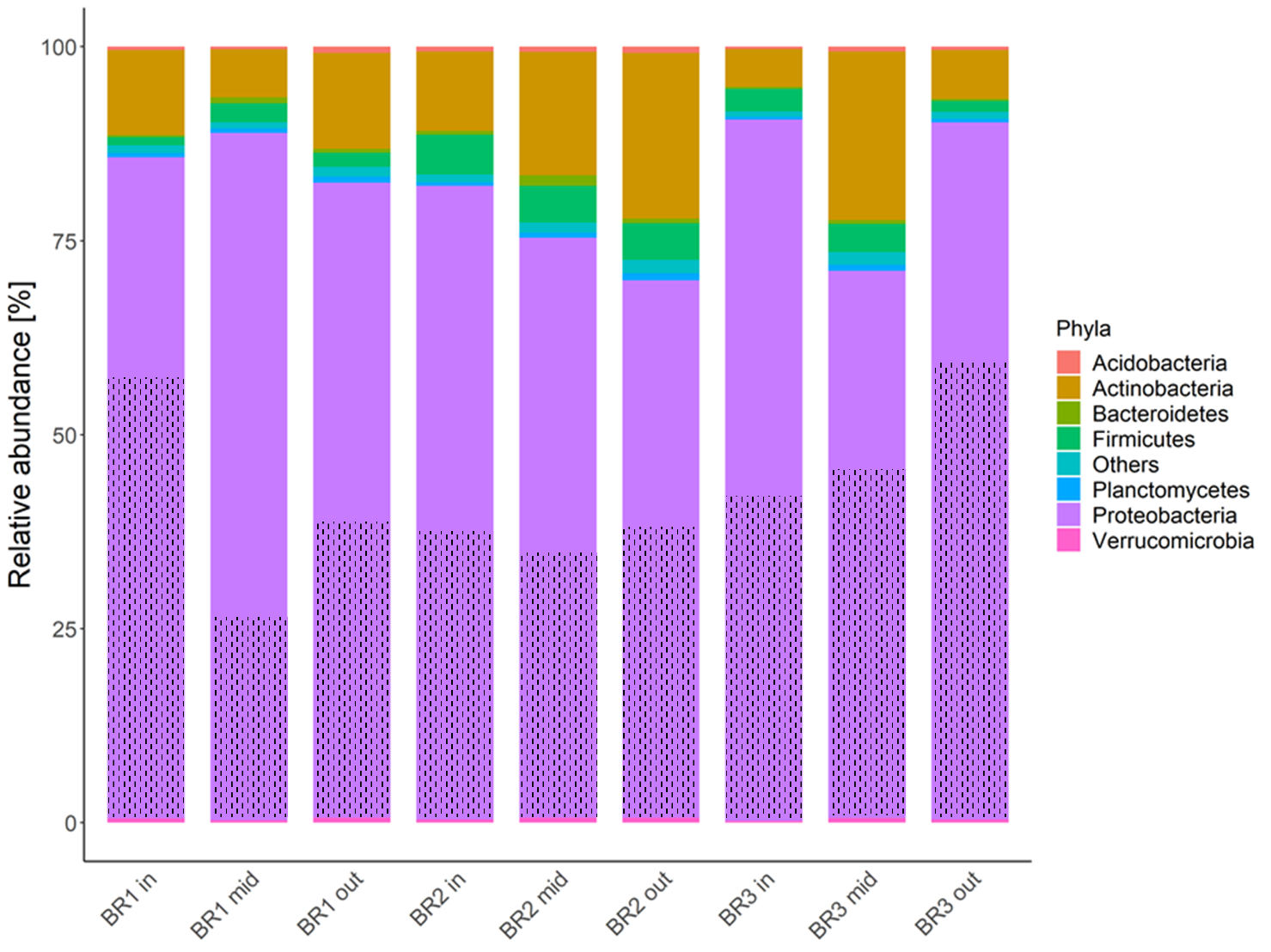


**Supplementary Figure 1.** Relative abundance of phyla in the metagenomics analyses of microbiomes from the bioreactors BR1, BR2, and BR3 at the zones of inlet (in), middle (mid) and outlet (out) of the agricultural drainage water flow. Dotted area of the bars for *Proteobacteria* indicate the contribution from the genus *Pseudomonas*.
